# Spiritual care from the perspective of family caregivers and nurses in palliative care: a qualitative study

**DOI:** 10.1186/s12904-023-01286-2

**Published:** 2023-10-26

**Authors:** Aslı Kurtgöz, Elif Keten Edis

**Affiliations:** 1 Department of Therapy and Rehabilitation, Sabuncuoglu Serefeddin Health Services Vocational School, Amasya University, Amasya, 05100 Türkiye; 2 Department of Nursing, Health Science Faculty, Amasya University, Amasya, Türkiye

**Keywords:** Family caregivers, Nurses, Palliative care, Spirituality

## Abstract

**Background:**

The palliative care period not only affects patients but also family caregivers in many ways. Palliative care units are places where the spiritual needs of family caregivers become important. According to a holistic care approach, palliative care nurses should determine the spiritual needs of family caregivers and help meet these needs.

**Objective:**

This study aims at exploring nurses’ and family caregivers’ experiences of spiritual care.

**Methods:**

A phenomenological study was designed in this qualitative research. A total of 10 nurses working in palliative care and 11 family caregivers participated in the study. Nurses’ experiences of delivering spiritual care and family caregivers’ experiences of receiving spiritual care were examined through the in-depth interviewing method on a one-to‐one basis. The data were examined using thematic analysis.

**Results:**

Four main themes were obtained by the data analysis: (I) Impacts of being in a palliative care unit; (II) Coping methods; (III) Importance of spirituality and spiritual care; (IV) Spiritual care. The results were presented according to the COREQ criteria.

**Conclusion:**

Although spiritual care is very necessary for family caregivers, it is not offered sufficiently due to nurse-and institution-related reasons. Palliative care nurses should determine the spiritual needs of family caregivers in line with the holistic care approach. Nurse managers should determine factors preventing nurses from offering spiritual care and create solutions for these factors. The lack of nurses’ knowledge about spiritual care should be resolved by providing continuous training and therefore, nurses’ competencies in spiritual care should be improved.

**Supplementary Information:**

The online version contains supplementary material available at 10.1186/s12904-023-01286-2.

## Introduction

Palliative care physically, psychologically, socially, and spiritually supports patients and family caregivers, thus increasing their quality of life [1]. Family caregivers are affected while delivering care to their patients and therefore, they need supportive care [2]. In the study of Chua et al. (2020), family caregivers were found to need more support than patients [3]. Considering this finding, the needs, and expectations of family caregivers in the context of palliative care services should be comprehensively evaluated and met [4]. The process of providing palliative care is a difficult experience that physically, psychologically, socially, and economically affects family caregivers. Furthermore, this process also spiritually affects family caregivers. Family caregivers experience some problems in spiritual matters such as finding meaning, uncertainty about the future, and hopelessness [5, 6].

Spirituality helps family caregivers to avoid emotional problems, be satisfied, feel strong and peaceful, find the meaning of their caregiver roles, as well as cope with their sadness and losses [7, 8]. A previous study reported that family caregivers with spiritual problems had higher levels of denial, anxiety, depression, difficulty in coping, and poor life quality [9]. In this regard, to improve family caregivers’ quality of life during the palliative care period, their spiritual needs should be identified and met. However, previous studies indicated that family caregivers could not receive sufficient spiritual care although they need [10, 11]. This finding highlights that spiritual care is an unmet need for palliative care family caregivers. Nurses are integral components of the spiritual care process for patients and family caregivers during end-of-life care [12]. Therefore, nurses are expected to be aware of individuals’ spiritual needs. To the best of our knowledge, no study has yet investigated the combined experiences of family caregivers of palliative care patients and palliative care nurses regarding spiritual care. This was the main focus of our study. It is believed that evaluating matters such as perceptions, needs, expectations, practices, and barriers related to spiritual care in palliative care from the viewpoints of care recipients (family caregivers) and care providers (nurses) will make a valuable contribution to the existing literature. In this regard, this study aims at exploring the experiences of family caregivers and nurses working in palliative care units regarding spiritual care.

## Method

### Study design

In this qualitative study, a phenomenological design was used to obtain in-depth information about nurses’ and family caregivers’ experiences of spiritual care.

### Setting and participants

The study was carried out with 10 nurses working in the palliative care unit (PCU) of a state hospital in Türkiye and 11 family caregivers in the same PCU. The purposive sampling method was used to determine participants. The nurses’ inclusion criterion was working in a PCU. The family caregivers’ inclusion criteria were set as being the most responsible family member for the patient’s care, being age 18 or older, and having no communicational problems (speaking, hearing, etc.). The number of participants was determined according to the principle of data saturation.

### Data collection

The data for this study were collected with face-to-face in-depth interviews using semi-structured interview quide between December 2022 and February 2023. The interviews were conducted by the author Mrs. E.K.E (Ph.D) in a private room in the PCU. The interview quide consisted of open-ended questions prepared by the researchers for this study (Suppl. file 1). Before the interviews, code names (N1, FC1, etc.) were assigned to the participants. The interviews were recorded using a voice recording device after receiving the participants’ consent. Each interview took approximately 15–50 min.

### Data analysis

The thematic analysis of qualitative data was structured according to Braun and Clarke’s (2006) six phases [13]. First, all interview recordings were listened to and transcribed. The obtained 21 written transcripts were repeatedly read by all researchers. In the second phase, codes representing meaningful statements were identified. Identical codes from different parts of the interviews were matched and combined. In the third phase, the codes were grouped under the belonging themes. In the fourth phase, themes obtained by each researcher were reviewed by all researchers. To simplify the themes, the researchers assessed whether each theme contained sub-themes. Sub-themes were identified for comprehensive and intricate themes, and codes were then grouped under related sub-themes. In the fifth phase, the themes and sub-themes were reviewed again by an independent researcher. According to the independent researcher’s suggestions, the themes and sub-themes were finalized. Lastly, four main themes and 11 sub-themes were obtained (Table 1). In the final phase, a research report was written selecting quoted participant responses about the themes (Table 1). Finally, the obtained results were reported according to the COREQ criteria.

### Trustworthiness

The validity and reliability of the data were obtained using the criteria proposed by Lincoln and Guba (1985) [14]. To assess the confirmability principle, interview recordings were listened to again and compared with the transcriptions. During the interviews, the participants’ statements were repeated in summary form by the interviewer to confirm whether they were understood correctly. For the dependability criterion, all interviews were conducted by the same researcher. All researchers have sufficient academic experience and certification in qualitative research. To ensure the transferability principle, the participants were selected using purposeful sampling. The participants’ statements were directly received, and no modifications were made. For credibility, the researchers examined the transcriptions independently. Furthermore, the themes and sub-themes were reviewed by an independent expert.

## Results

The nurses’ mean age was 35.8 ± 6.73 years (27–44), their working experience in the PCU ranged from 5 months to 7 years, and their overall work experience ranged from 5 to 23 years. 54.5% of the family caregivers were female, with a mean age of 43.09 ± 10.11 (27–54), while their patients’ hospitalization time ranged from 3 to 92 days. The family caregivers’ educational levels were as follows: primary (54.5%), high school (27.3%), vocational school (9.1%), and college (9.1%).

### Theme-1: impacts of being in a palliative care unit

Within the theme “Impacts of being in a Palliative Care Unit,“ four sub-themes were identified namely, “Impacts on physical health”, “Psychological, emotional, and spiritual impacts”, “Economic impacts” and “Impacts on personal development and life”.

### Impacts on physical health

Family caregivers expressed feelings of fatigue, struggles in providing care, and sleep deprivation while accompanying their patients in the PCU (Table 1, FC9).

### Psychological, emotional, and spiritual impacts

Family caregivers conveyed feelings of sadness due to their patients’ suffering and their own inability to care for their families and/or children during this period. Consequently, they stated that they experienced overwhelming and intense stress, as well as feelings of helplessness, exhaustion, depression, and anger (Table 1, FC2 and FC6).

Also, nurses expressed that their work in the PCU had various effects on them. They highlighted that the predominant emotion they often encountered while working in the PCU was sadness. Nurses stated that witnessing death, especially the death of young patients makes them very sad (Table 1, N1).

### Economic impacts

Some family caregivers stated that the treatment process of their patients economically affected them. Furthermore, employed family caregivers mentioned that the care process also affected their work life. Some family caregivers took leave from their jobs to be with their patients; whereas some others tried to continue both caregiving and work responsibilities simultaneously (Table 1, FC5).

### Impacts on personal development and life

The majority of the nurses stated that their work in the PCU contributed to their personal development and altered their outlook on life. A majority of the nurses commented that working in a PCU provided them with some positive outcomes such as being sensitive, learning to be grateful, gaining a positive perspective in life, and developing empathy skills (Table 1, N4, N3 and N9).

### Theme-2: coping methods

Based on the nurses’ statements, coping methods with the effects of being in the PCU were grouped in two sub-themes namely, “Avoidance” and “Acceptance”. Similarly, in consideration of the family caregiver’s expressions, their coping methods were grouped under two sub-themes: “Religious coping methods” and “Social support and sharing”.

The nurses stated that they often used avoidance and acceptance to cope with the problems being in the PCU. Some of the nurses stated that they abstain from establishing emotional bonds with patients to avoid being affected by working in the PCU. A majority of the nurses said they accepted that death was inevitable and got used to patient deaths (Table 1, N1).

On the other hand, the family caregivers stated that they used religious coping methods frequently and received social support to cope with difficulties. the family caregivers commented that they used some methods including praying, seeking refuge in God, being patient, getting support from relatives, and talking to relatives of other patients (Table 1, FC3 and FC1).

### Theme-3: importance of spirituality and spiritual care

The nurses stated that spirituality and spiritual care provide family caregivers with motivation, a feeling of confidence and resilience, as well as help them to relieve, accept, and cope (Table 1, N8 and N1).

Family caregivers emphasized that spirituality and spiritual care help them be patient and bear with, feel relieved and safe (Table 1, FC4 and FC11).

### Theme-4: spiritual care

Within the theme of “Spiritual Care,“ spiritual care practices offered by PCU nurses to family caregivers, the spiritual care needs of family caregivers, and the factors hindering nurses from providing spiritual care were identified from the viewpoints of both nurses and family caregivers.

### Spiritual care practices

The spiritual care practices provided by nurses for family caregivers were allowing them to spend time with their patients, helping them to pray, showing a friendly approach, and directing them to the spiritual care department (Table 1, N9).

Some of the nurses stated that they direct family caregivers to pray according to their own faith (Table 1, N7 and N8).

On the other hand, family caregivers stated that the spiritual care practices they received in the PCU were the friendly approach of nurses and allowing them for praying (Table 1, FC1 and FC4).

### Needs

According to the nurses, the needs of the family caregivers in the PCU are speaking, hoping, and getting informed about their patient’s status (Table 1, N4 and N3).

According to the family caregivers, their needs are a private room in the PCU for praying, continuous presence of Muslim religious officers, friendly attitudes of nurses, dialogue with and being relieved by the nurses, and hopeful speeches of doctors and nurses (Table 1, FC1 and FC9).

### Barriers

According to the nurses, barriers to providing spiritual care were workload and large numbers of patients, insufficient number of nurses, lack of time, unsuitable physical conditions, and doctors’ attitudes (Table 1, N6, N9 and N10).

Some of the nurses commented that they feel inadequate in spiritual care, they lack information and want to receive train (Table 1, N1).

**Table 1:** Themes, sub-themes, and representative quotes

Themes and Sub-themes	Representative quotes
Theme 1: Impacts of Being in a Palliative Care Unit	
*Impacts on physical health*	“Being here seriously affects my life. I have three children. I can’t care for them because I’m here. There is no one else to help me. I do everything myself. It is very difficult to lift my patient and turn her around only by myself. I could not sleep for five nights. All these make me very depressed.” *(FC9)*
*Psychological, emotional, and spiritual impacts*	“I’m psychologically exhausted. I’m here for three full months. What I hear (cries of patients, sounds of devices connected to patients, cries of family caregivers) still bothers me…” *(FC2)*
	“In fact, I reside in another city, but I’ve left my wife and children to be here with my mother. I had to make this decision due to my deep love for her. I am now devoted to her. Witnessing my mother’s suffering is truly distressing for me.“ (*FC6)*
	“When I first started working here, I was affected by witnessing so many patient deaths. It’s easier to accept the death of elderly patients. However, it is very difficult for us to accept the death of young patients, it is so sad.” *(N1)*
*Economic impacts*	“I am the sole breadwinner in our home; nobody else is employed. While working, I am also the one accompanying my mother here. There isn’t anyone else in the family capable of taking on these responsibilities (meaning accompanying their patient). My mother’s illness has also affected us financially. So, I have to work and earn money, but managing both tasks is very challenging.“ *(FC5)*
*Impacts on personal development and life*	“Working here showed me that life is meaningless. I’ve recognized that it’s pointless to worry about too little things. I’ve understood that there was no need to be sad and offend (others).” *(N4)*
	“Working here has shifted my perspective. For example, I realized that one shouldn’t give excessive importance to everything in life. Eventually, life is too short. I learned to be sensitive toward my family and others. If I wasn’t working here, probably I wouldn’t be so sensitive.” *(N3)*
	“Working here has changed me in every way. My empathy skills have improved. Here, I feel the existence of death better. This changed my perspective on life.” *(N9)*
Theme 2: Coping Methods	
*Avoidance*	“Patients become one of us because they stay (in the hospital) for a long time. Over time, an emotional bond develops between us. Once, I was very saddened by the death of a young patient. After that, I made a decision that I would never establish an emotional bond with anyone here. Now I’m thinking we’re all going to die one day. So, I accepted the existence of death.” *(N1)*
*Acceptance*	
*Religious coping methods*	“Everyone seeks a refuge when faced with a challenge. My refuge is my faith. If my religious beliefs were not strong, I would not have been able to cope with what I went through.” *(FC3)*
*Social support and sharing*	“I speak relatives of other patients here. We pour our hearts out to each other and relax. They pray for me, and I pray for them.” *(FC1)*
Theme 3: Importance of Spirituality and Spiritual Care	“Spirituality helps family caregivers to accept more easily. For example, a family caregiver with a weak faith might be more aggressive. They may cause distress for both themselves and the patient. However, when family caregivers have religious faith, this difficult period becomes easier. They would not cause any difficulties either for themselves or for the patient.” *(N8)*
	“Particularly the patients’ children cannot accept that their loved one is close to death and may pass away at any time. Providing spiritual care is crucial to gradually help them adapt to this process and come to terms with it. Moreover, spirituality significantly eases the coping process for the relatives.” *(N1)*
	“In my opinion, spirituality comes first. Without spirituality, one becomes irritable and shows no patience when encountering troublesome events. Spirituality brings endurance and patience. This is why I believe it’s very important.” *(FC4)*
	“For me, spirituality is the value and support that my family and acquaintances show me. As they support me, everything gets easier. I feel safe and relieved.” *(FC11)*
Theme 4: Spiritual Care	
*Spiritual care practices*	“Here, we don’t implement a visitor restriction. They pray near their patient and read Quran aloud. They are free to do whatever they want to do according to their beliefs. We give them all kinds of support accordingly. Also, I believe a smile is good for everyone. Talking with a smile is relieving for both patients and their relatives.” *(N9)*
	“I say to family caregivers ‘pray and read the Qur’an instead of crying.” *(N7)*
	“Family caregivers don’t know what to do. And we say to them ‘pray, the only thing you can do is praying’. We direct them to pray to help them get used to the death of the patient.” *(N8)*
	“They treat us with a smile. They always ask me how I am” *(FC1)*
	“There are Quran and prayer rugs in the cabinets here. There is a prayer room downstairs. I go there for praying.” *(FC4)*
*Needs*	“They have some religious needs. Family caregivers want to read Qur’an near their dying patient. Thus, they feel relieved. They also get relieved as we speak to them.” *(N4)*
	“Relatives are literally looking us in the eye. They expect us to be close to them and talk to them. They especially expect us to say something hopefully and good about their patients.” *(N3)*
	“I try to do my prayer in the patient room on a piece of cardboard. I wish there was a praying room in this unit. Additionally, it would be nice if the nurses and doctors didn’t say desperate words. When they say desperate words, one gets demoralized. They may talk more supportive.” *(FC1)*
	“If nurses smile and ask to us ‘how are you?’, this would give us morale. We have been in the hospital for a long time. No one supported us or even spoke to relieve us.” *(FC9)*
*Barriers*	“Spiritual care is very important in palliative care. However, in the current conditions, we cannot provide both patient care and spiritual care to the patients/relatives at the same time. High workload affects us. There are 10 patients per nurse on each shift. So, we can only do our routine work, treatment. This is why I don’t have either enough strength or time to offer spiritual care.” *(N6)*
	“There are patient rooms in this unit that never receive sunlight. Patients stay hospitalized for months in enclosed rooms without any natural light. The family caregivers also accompany their patients in such an environment for months. I believe that patients should be able to spend their final moments in comfort in every aspect. For instance, if this unit was on the ground floor and if we had a door leading to the garden, and if we could take the patients to the garden, it would bring significant relief for them.” *(N9)*
	“Here, there are two approaches. One of them is treatment-oriented, and the other is an approach including less treatment but spiritual care. If the doctor is treatment-oriented, we can only provide treatment. We don’t have time to do anything but treatment. However, some doctors concerning about how to relieve the patient in their final time. For such doctors, we can offer spiritual care. In short, we can offer spiritual care depending on the doctor.” *(N10)*
	“I wish I would have the competency and training for spiritual care. The only thing we could do is to direct them to pray, that’s all. The practices we know, and implement are not professional, just our daily life observations. I wish I would have more knowledge.” *(N1)*

## Discussion

After the diagnosis of a life-threatening illness in a family member, family caregivers face many difficulties during treatment and end-of-life care [15]. Therefore, the care period is a difficult experience that affects family caregivers in many ways. The current findings showed that family caregivers face several difficulties during palliative care such as sadness, exhaustion, stress, helplessness, anger, depression, problems in work life, difficulty in caregiving, fatigue, problems in caring for their own family, and sleeplessness. Similar to our findings, previous studies also indicated that family caregivers experience many difficulties including anxiety, sadness, financial difficulties, problems in maintaining family relationships, exhaustion, difficulty in caregiving, emotional exhaustion, fatigue, depression, and fear [10, 16–20]. Furthermore, our findings revealed that witnessing difficult situations during the palliative care of their patient and taking care of the patient have many versatile and negative effects on family caregivers. This finding indicates the need for considering family caregivers from all aspects and meeting their needs.

It was found that the family caregivers who participated in our study used some coping methods such as praying, seeking refuge in God, being patient, receiving support from relatives, and speaking with relatives of other patients about the problems they encounter. Literature findings indicate that religious belief relieves the family caregivers during the caregiving period [21]. Rocha et al. (2018) highlighted that family caregivers do religious rituals such as praying, reading Bible, and relying on God as well as sharing their experiences with others to cope with the problems they face in the PCU [22]. It was emphasized that establishing a religious and spiritual environment in palliative care is important for supporting family caregivers and strengthening individuals’ coping mechanisms [23]. According to our findings, to support family caregivers’ coping skills, they should be provided with a proper environment to do their religious rituals and allowed to share their experiences.

Our findings showed that spiritual care helps family caregivers to be patient and bear with, feel relieved and safe. Consistent with our results, previous studies indicated that spirituality helps family caregivers to cope with difficulties and reduces their emotional burden [9, 24]. Furthermore, the results of a study conducted in South Korea revealed that unmet religious and spiritual needs of family caregivers increased their care burden [25].

Spiritual care allows supporting individuals and identifying their needs during important transitional periods including illness and loss [26]. According to our results, family caregivers received a friendly attitude from the nurses, and they were allowed to pray within the scope of spiritual care. Moreover, it was determined that some nurses directed family caregivers to pray according to their faith within the scope of spiritual care practices. Our findings revealed that nurses focused on mostly religious activities for spiritual care, considered the concepts of religion and spirituality the same, and therefore, were in a misconception. The literature highlights that spiritual care should not be limited to only religious practices but also include promoting hope, empathizing, and meeting individuals’ other inner existential needs that help them find meaning in their circumstances [27]. Furthermore, nurses should follow ethical guidelines while offering spiritual care and should avoid imposing their personal beliefs or practices on others [28].

The study findings indicated that there are some unmet spiritual needs of family caregivers. Studies on this subject reported that family caregivers need spiritual care, to feel hopeful, to be prepared for their patient’s death in palliative care, and therefore, expect nurses to establish a relationship with them based on compassion, love, sincerity, and empathy [22, 29, 30]. Meeting the spiritual needs of family caregivers is an important part of palliative care [31]. Accordingly, nurses should be aware of the spiritual needs of family caregivers while offering care. In future studies, exploring the underlying factors that hinder palliative care patients and family caregivers from fulfilling their spiritual care needs are believed to provide insights for developing solutions to address these barriers.

According to our findings, working in the palliative care unit affected nurses in various ways. It was found that the nurses experienced intense sadness especially, due to witnessing the death of young patients. Similarly, the results of a qualitative study carried out in China showed that nurses are more saddened by the death of their young patients [32]. Elderliness is a physiological phenomenon and represents the last developmental period of human beings [33]. This situation makes nurses think the death of the elderly is inevitable, thus they can accept the death of elderly patients more easily. However, the death of young patients may cause nurses to be sadder because nurses may put themselves in young patients’ shoes and their death trigger nurses to think about their own death. This finding indicates that the emotional burden on nurses resulting from working in the PCU can personally and professionally affect nurses. This emotional burden can also negatively impact nursing care. Furthermore, the frequent experience of patient death can also influence nurses’ spirituality in various ways. Therefore, we believe that all PCU staff, especially inexperienced health professionals, require professional support to assist them in protecting themselves from the potential negative effects that can arise from working in the PCU. We recommend that future studies employing diverse designs thoroughly explore the emotional, psychological, and spiritual impacts of working in the PCU on nurses and other team members. Therefore, it is believed that such research will contribute to identifying the requirements of PCU staff and the development of strategies to address these needs.

The nurses stated that working in the PCU provided them with some outcomes such as gaining a positive perspective in life and improved empathy ability. Okçin (2019) found that the experiences of nurses working in a PCU have some positive outcomes including contributing to their professionalization processes, better emotional control, and making sense of life [34]. Accordingly, it can be argued that witnessing patient deaths frequently directs nurses to spiritual considerations such as questioning and seeking the meaning of life and death.

According to the results, some reasons including too much workload and lack of time prevent nurses from offering spiritual care. Consistent with our findings, other studies also determined the reasons preventing nurses from delivering spiritual care were lack of knowledge about spiritual care, excessive workload, lack of experience, low motivation, lack of human resources, lack of time, lack of special room for spiritual care, lack of religious and spiritual facilities and resources in the hospital [35–38]. Under these demanding working conditions, nurses might face challenges in delivering high-quality holistic care. This situation could potentially lead nurses to prioritize physical care while inadvertently neglecting spiritual care. Accordingly, apart from enhancing nurses’ working conditions, we believe that hospital administrators and nurse leaders/managers should formulate practices and strategies aimed at removing barriers to delivering spiritual care. Additionally, providing spiritual care in collaboration with spiritual care professional is also very important. Therefore, to adequately address the spiritual needs of patients and family caregivers in collaboration with nurses- spiritual care professionals, it’s essential to ensure an adequate number of spiritual care professionals within hospitals. This will contribute to achieving the desired level of spiritual care.

Literature suggests increasing the competencies of healthcare personnel to address and support patients and family caregivers psychosocially and spiritually [39]. However, studies carried out in various countries reported that the spiritual care competency of health personnel was below the desired level, nurses feel inadequate in delivering spiritual care and need training [35, 40–42]. Similarly, in this study, we determined that nurses perceive themselves as inadequate in delivering spiritual care and want to receive training. Accordingly, nurses should be supported to enhance their knowledge and competence in spiritual care.

## Conclusion

In conclusion, our findings indicate that spiritual care is an important need for family caregivers, but it is not delivered at the desired level. Therefore, we recommend nurses should be provided with relevant competencies through training about the importance of spiritual care and how to implement it. Furthermore, nurses should be aware that spirituality and religion are concepts with different meanings and should not think that spiritual care is only helping to do religious rituals. Nurses should avoid directing individuals according to their own religious beliefs while delivering spiritual care. Finally, we suggest that the factors preventing nurses from delivering spiritual care should be identified and eliminated by manager/leader nurses and hospital managers.

### Limitations

This study covers only family caregivers and nurses working in the PCU of a state hospital in Türkiye. Due to the cultural characteristics and personal palliative care experiences of the participants, the obtained results cannot be generalized to all nurses and family caregivers.

### Electronic supplementary material

Below is the link to the electronic supplementary material.

Supplementary Material 1

## Data Availability

The interviewees discussed identifying details in their open-ended responses, concerning themselves, patients, family caregivers, nurses and other providers (institution managers etc.), and the process of de-identification would be difficult and complicated, so that the data are not publicly available on any websites at the moment. The datasets/written transcripts used and/ or analyzed during the current study are available from the corresponding author on reasonable request.

## Abbreviations

COREQ: consolidated criteria for reporting qualitative research
FC: family caregiver
PCU: palliative care unit
N: nurse
