# Supplementary material for: Spiritual care from the perspective of family caregivers and nurses in palliative care: a qualitative study
Source: BMC Palliat Care. 2023 Oct 26;22:161. doi: 10.1186/s12904-023-01286-2 (PMC10601296; doi:10.1186/s12904-023-01286-2)
Supplement: Supplementary file 1 — Supplementary Material 1 [file 12904_2023_1286_MOESM1_ESM.docx]

**Interview quide**

| Interview questions for nurses:  In your opinion, in what ways working in PCU has affected you?  What do you do to deal with these issues that affect you?  Could you please tell us about the importance of spiritual care in palliative care?  In your opinion, what are the spiritual care needs of family caregivers in PCU?  What services do you offer to family caregivers within the scope of spiritual care?  What are the barriers to you delivering spiritual care in PCU? |
| --- |
| Interview questions for family caregivers:  In your opinion, in what ways being in PCU as a family caregiver has affected you?  What do you do to deal with these issues that affect you?  Does spirituality have an impact on you during the disease period of your patient?  Do you need spiritual care in PCU?  Do you receive spiritual care from nurses in PCU?  What kind of spiritual care would you like to receive from nurses in PCU? |
